# Trials of Intervention Principles: Evaluation Methods for Evolving Behavioral Intervention Technologies

**DOI:** 10.2196/jmir.4391

**Published:** 2015-07-08

**Authors:** David C Mohr, Stephen M Schueller, William T Riley, C Hendricks Brown, Pim Cuijpers, Naihua Duan, Mary J Kwasny, Colleen Stiles-Shields, Ken Cheung

**Affiliations:** ^1^ Center for Behavioral Intervention Technologies (CBITs) Northwestern University Chicago, IL United States; ^2^ Office of Behavioral and Social Sciences Research National Institutes of Health Washington DC, DC United States; ^3^ Department of Psychiatry Northwestern University Chicago, IL United States; ^4^ Department of Psychology and Pedagogy Vrije Universiteit Amsterdam Netherlands; ^5^ Department of Psychiatry Columbia University New York, NY United States; ^6^ Department of Preventive Medicine Northwestern University Chicago, IL United States; ^7^ Mailman School of Public Health Columbia University New York, NY United States

**Keywords:** mHealth, eHealth, clinical trials, methodology

## Abstract

In recent years, there has been increasing discussion of the limitations of traditional randomized controlled trial (RCT) methodologies for the evaluation of eHealth and mHealth interventions, and in particular, the requirement that these interventions be locked down during evaluation. Locking down these interventions locks in defects and eliminates the opportunities for quality improvement and adaptation to the changing technological environment, often leading to validation of tools that are outdated by the time that trial results are published. Furthermore, because behavioral intervention technologies change frequently during real-world deployment, even if a tested intervention were deployed in the real world, its shelf life would be limited. We argue that RCTs will have greater scientific and public health value if they focus on the evaluation of intervention principles (rather than a specific locked-down version of the intervention), allowing for ongoing quality improvement modifications to the behavioral intervention technology based on the core intervention principles, while continuously improving the functionality and maintaining technological currency. This paper is an initial proposal of a framework and methodology for the conduct of trials of intervention principles (TIPs) aimed at minimizing the risks of in-trial changes to intervention technologies and maximizing the potential for knowledge acquisition. The focus on evaluation of intervention principles using clinical and usage outcomes has the potential to provide more generalizable and durable information than trials focused on a single intervention technology.

## Introduction

### Background

Behavioral intervention technologies (BITs) employ technologies, such as mobile phones, tablets, computers, sensors, and other tools to support behavior change related to health, mental health, and wellness. Mobile health apps, treatment and prevention websites, sensors used in activity trackers, and smartwatches are common examples [1]. The term BIT is used, rather than eHealth or mHealth, as these terms can reflect a much broader area of medicine and informatics not necessarily focused on behavior change [2]. In practice, BITs change and evolve over time. As anyone who has installed an app knows, their life on a device evolves through a steady stream of updates. Many of these updates are bug fixes, operating system upgrades, or changes to support the emergence of new devices. However, some changes alter the content and functionality and are intended to modify or improve the user’s experience and the benefit they receive.

BITs change to harness affordances provided by the rapidly changing technological environment, such as improving computing power, leveraging new data capture and user interface functions, and growing capacity for data transmission [3]. The expectations and culture of BIT users are rapidly changing. When developers of a BIT observe that a feature used in other apps has become popular, they often add similar functionality to respond to the expectations of their customer base. For example, social networking and peer-to-peer messaging is a common feature in many recently developed BITs due to the popularity of social networking tools such as Facebook, Twitter, etc. Failing to meet changing user expectations relegates a BIT to increasing irrelevance to users. There have been increasing calls for methodologies that allow for continuous quality improvement through more rapid incorporation of changes and accumulating knowledge in the context of trials [4-6]. The purpose of this paper is to propose adaptations of traditional randomized controlled trial (RCT) methodology that can support evaluation of BITs.

### The Purpose of a Randomized Controlled Trial

Modern RCT methodologies in medicine were developed to evaluate pharmacological agents that are not intended or expected to be modified frequently. To respond to early critiques that psychological interventions have little effect [7], rigorous methodology was developed, often using principles from pharmacological trial design, to evaluate psychological and behavioral interventions. Among the many innovations and adaptations, psychological treatments were standardized through manualization, therapist training, supervision, and fidelity monitoring to maximize internal validity [8]. These rigorous methods of “locking down” a psychological treatment contributed to a vast literature supporting the efficacy of psychological and behavioral interventions, as well as broad acceptance of their clinical value. However, it is doubtful that even with these methodologies, behavioral interventions are truly locked down to the degree a pharmacological agent is during a trial. And once moved to real-world clinical practice, they are rarely implemented in the same manner as in the RCT [9,10]. The application of RCT methodology developed for pharmacotherapies, translated for behavioral and psychological interventions, to BITs is even less appropriate.

There are two primary problems with locking down a BIT in an RCT. First, the length of time required to conduct an RCT is often not consistent with the rapidly changing technological environment [11] and user expectations. In some cases, trials of BITs can be conducted rapidly, however, many times trials must extend over longer periods of time. This is especially true when adequately powering a trial necessitates extended participant recruitment. The number of participants required to power a traditional RCT makes sense when the resulting intervention (if found successful) will have a long shelf life and benefit many users over time. In RCTs for BITs, however, this is rarely the case. RCTs often test early versions of BITs that often have to undergo revisions prior to implementation due to factors not under the developers’ or researchers’ control, such as changes in technological environment and contextual factors (eg, perceived attractiveness and usefulness of a BIT), which can impact use patterns or outcomes if not addressed [6]. Thus, locking down a BIT means that the information gained from a trial might not be useful for the implementation, even if this takes place shortly after the trial period.

Second, and perhaps more importantly, new information is gained within the trial that can be used for quality improvement [12]. Even with careful user-centered design and pilot testing, problems in the BIT design that impact the user’s ability or willingness to perform tasks are often uncovered when it is deployed at scale for the full trial. As these deficiencies are discovered, locking down the BIT and refusing to improve it may undermine the chances of success, waste effort and resources, and compromise the relevance in the knowledge gained, if any.

Thus, BIT researchers are left with a difficult choice. They can (1) continue to investigate the locked-down version to maintain internal validity (with increasing understanding of the problems in the BIT and its growing irrelevance), (2) make changes to the BIT but not report them (reducing the reliability of the scientific literature and limiting the ability of other researchers to build on the work appropriately), or (3) make changes to the BIT and report them (raising the question “What is being tested?”).

Current RCT methodology is based on the lock-down model, well suited to determining if a fixed intervention is more efficacious than a control condition. However, given the rate of technological advancement and changes in user expectations, we argue that testing a fixed BIT often has very little public health value because the shelf life of that fixed BIT is often relatively short. We argue instead for allowing modification to BITs if these changes are reported. The question of what is being tested can be addressed by specifying the principles (model or rules) underlying the BIT and conceptualizing the trial as testing those principles, and not the BIT itself.

### Trials of Intervention Principles

We argue that trials of BITs should be viewed as experiments to test principles within that BIT that can then be more broadly applied by developers, designers, and researchers in the creation of BITs and the science behind technology-based behavioral intervention. As such, we refer to these trials as “Trials of Intervention Principles” (TIPs), as they test the theoretical concepts represented within the BIT, rather than the specific technological instantiation of the BIT itself. Similar logic has been applied in other instances of interventions to identify the elemental components contained within the interventions, to improve the ability to generalize across trials, and to guide intervention selection, creation, and evaluation [13-15]. We define a principle as a model or set of rules that defines how a group of behavioral strategies is instantiated in a BIT and how the use of that BIT leads to an outcome. For example, a trial might evaluate an app that aims to increase exercise by including functionality that is intended to support goal setting, monitoring, and feedback. A TIP would allow for optimization of the BIT in the service of testing the principle. For example, the monitoring tools might prove less than ideal and thus evolve over time, moving from text-based free entry to selecting from a list, to passive sensing. Some of these evolutions might improve usability and thereby outcomes (eg, shifting from user-initiated entry to passive sensing could reduce user burden, thereby providing more feedback and increasing value to the user). On the other hand, improving usability could be detrimental, either because the improved usability in one part of the BIT creates problems in another (eg, shifting from user-initiated entry to passive sensing might not be able to sense certain activities, like bicycling, thus frustrating the user and limiting their subsequent use of the BIT), or because improved usability interferes with behavioral aims (eg, shifting from active behavioral logging to passive sensing may reduce agency or remove opportunities for the user to notice and learn about their behaviors). A TIP would allow for these iterations to improve the usability of the BIT based on use data, while continuing to test the principle that using a mobile phone app to promote goal setting, monitoring, and feedback to increase exercise is effective, based on the primary outcome data.

In light of this, TIPs test a set of intervention principles that define an underlying rule, model, or mechanism of action on which a BIT impacts its target outcome. The principles being tested may be based on clinical theory and/or methods of using technology to interact with users. TIPs require a methodology that provides guidance on operationalizing and analyzing these principles. Below we describe a framework for describing and operationalizing those principles, considerations related to making changes to a BIT, and evaluation methodologies.

## The Behavioral Intervention Technology Model

### Overview

The principles to be tested must be defined and operationalized a priori to provide clarity to the aims, support change decisions during the trial (eg, defining what can and cannot be changed), as well as provide a method of documenting and reporting changes in a consistent manner. A framework for the definition of BIT principles can support investigators in characterizing these principles.

**Figure 1:**
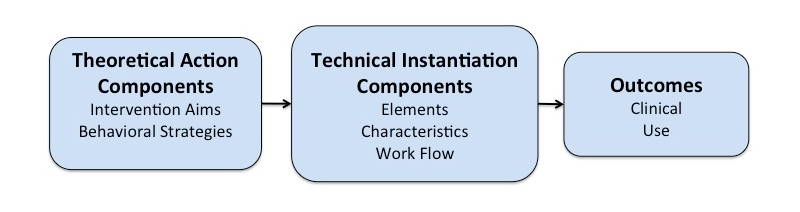
The BIT Model.

The BIT Model, shown in Figure 1, answers the questions “why”, “how”, “what”, and “when” of BIT development and includes two broad levels: (1) a theoretical action level, which reflects the intentions of the developer or researcher, and (2) an instantiation level, which reflects the technological implementation [16]. This model has been used to design and characterize intervention technologies [17].

### Theoretical Action Components

The theoretical level includes two components: (1) intervention aims (note that in contrast to the BIT Model paper [16], the term “intervention” here refers to the entire treatment package), which are *why* the BIT exists, and (2) behavioral strategies, which are conceptually *how* the BIT will achieve those aims.

### Intervention Aims

The intervention aims (“why” the BIT exists) reflect the fundamental intentions of the developer. These aims commonly include both explicit clinical aims (which may include preventive or well-being aims), such as weight reduction or reduction of depressive symptoms, as well as usage aims, which are sometimes implicit, such as expected frequency of use or the use of specific intervention elements. The clinical aims often have sub-aims. For example, a BIT that aims to reduce weight might aim to reduce caloric intake and increase physical activity. A BIT for depression may have sub-aims such as increasing positive activities and teaching cognitive restructuring. In general, the primary intervention aims, and frequently the sub-aims, are not changeable and are reflected in the assessment of primary outcome. Changing the primary aims of the BIT would require a new trial.

While clinical aims are most often mirrored by the primary outcome measurements, usage aims are more often expectations that the developer has regarding the level of use necessary for clinical effectiveness. While usage aims are not always highly correlated with outcome [18], they are often critical expectations, as they often provide early indicators of possible design flaws and may be used as indicators of the need for potential changes to the BIT.

### Behavior Change Strategies

Behavior change strategies are the methods used to attain intervention aims and are grounded in models and theories of how behavior change occurs and is maintained. For example, Michie has developed a well-grounded taxonomy of such change strategies [19,20] such as education, goal setting, monitoring, feedback, or motivational enhancement. Chorpita et al have similarly identified common practice elements among various behavioral and mental health interventions creating 26 codes in one instance and 47 in another [13,14].

Behavioral strategies are often key principles being tested in trials of apps (although this may not be true for trials with a human computer-interaction focus) and thus usually cannot be changed or eliminated during a trial. A decision to do so would prevent the investigator from being able to draw conclusions from the results of the trial.

### Instantiation Components

#### Overview

The instantiation components include (1) BIT elements (“what” is delivered), (2) characteristics of those elements (“how” they are delivered), and (3) workflow (“when” they are delivered). In many cases these components can be tracked through use data. These three instantiation components are operationalized in the hardware specifications and software programming code of the BIT.

#### Elements

BIT elements reflect “what” is provided to the user. The elements are the distinct objects of a BIT intended to implement the behavior change strategies, which in turn support the user in achieving the clinical and usage aims. By BIT elements, we mean the actual technical instantiations present in the BIT. For example, a content delivery element might provide the user with information. Notifications are individual messages pushed to the user, such as text messages, emails, or within app notifications. Logging elements allow users to enter information. Reports and visualizations are reflections of data collected by the BIT that are provided back to the user (eg, calendars, calorie counts, thought records), often used to instantiate behavioral strategies such as feedback. Thus, the BIT elements are the aspects of the BIT with which the user actually interacts.

Some elements in a BIT may be part of the principles being tested, and others may not. For example, an investigator may be interested in testing one or more methods of logging tools for behavior, making these part of the principles being tested. Other elements may be included but are not of scientific interest to the study. For example, reminders may be included to support use but may not be part of the principles being tested. In this case, the investigator could not eliminate the logging elements but might be able to alter some of the characteristics or workflow within the parameters of the principles being evaluated, such as adding reminders to cue logging activities. However, the investigator would have much greater latitude in altering, adding, or eliminating the reminder elements, as long as the alterations did not introduce a new principle that might compete with the original principles.

#### Characteristics

Characteristics are “how” the elements are deployed. Elements can be considered objects, while characteristics are attributes of those objects. For example, an informational element may have a variety of characteristics, such as text, video, or audio content. That content may be simple or more complex. Form and esthetics are also characteristics of the elements. Elements may be personalized using characteristics to fit the demographics, language, or culture of the individual user.

In general, behavioral scientists have been less concerned with the characteristics, other than to make the BIT more usable and attractive to the user. However, in some instances the characteristics of BIT elements are the principle being investigated, for example, when evaluating the utility of personalized applications relative to static ones [21]. In those circumstances, substantive alterations to those characteristics under investigation may harm the trial.

#### Workflow

Workflow reflects “when” specific elements of a BIT are delivered. Most BITs are designed for repeated interactions over an extended period of time. The workflow determines when specific sets of elements are delivered, the sequence of the delivery, and the length of the intervention. User-defined workflows contain no coded rules, allowing the user access to all intervention elements and content. Thus, the user decides the sequence and timing of their use. Many BITs set conditions for when specific elements are made available. Time-based rules define the release of an intervention element based on the passage of time. For example, Web-based treatments modeled on standard face-to-face treatments sometimes release new content on a weekly basis [22,23]. Event-based rules define the release of elements based on the criteria detected by the intervention, such as the completion of a task or the detection of a user state. Just in time interventions may use complex workflows that use combinations of user characteristics, use data, detected events, and time to determine the delivery of intervention components [24].

Workflow as a principle has not received much attention in BIT research. However, workflow may impact principles that are being evaluated. For example, shifting notifications from fixed time-based to event-based may change the underlying behavior change strategy, as a notification triggered by behavior may be a form of feedback. Thus, changes to workflow as well as other theoretical and instantiation components may introduce factors that researchers did not anticipate.

## Behavioral Intervention Technology Model in the Trials of Intervention Principles Framework

### Overview

**Figure 2:**
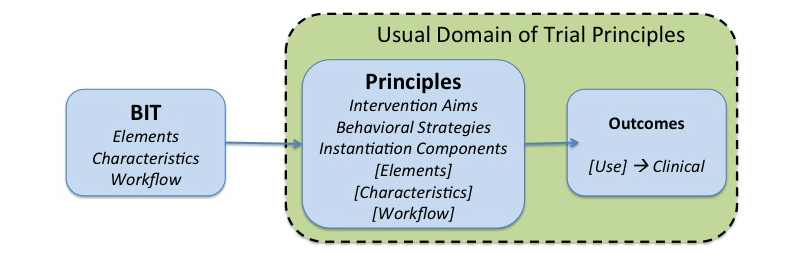
BIT Model in the context of TIPs.

Figure 2 places the BIT Model into the context of TIPs. Aspects included within the dotted box are the main focus of the TIP and therefore should not be changed during the course of the trial. The box on the left, labeled “BIT”, represents the instantiation features of the BIT, which define the intervention as deployed.

The central box represents the principles being tested in that deployment, which must always have an intervention aim and cannot change over the course of a trial. BITs designed by clinical or public health researchers almost always are based on some set of behavioral strategies, which often are also part of the intervention principles. Instantiation features (ie, elements, characteristics, or workflow) are represented in brackets because these may or may not be part of the principles of trials conducted by researchers, but often are the focus of trials with an engineering or human computer interaction focus. Instantiation components that are listed as principles may not be modified in the BIT during a trial. To the degree that those instantiation components are not part of the principles, or their alteration would not affect any of the principles, adaptation could be considered.

The outcomes box on the right represents the measurement of outcomes. The intervention outcomes generally also should not change. Use data (eg, use of a specific BIT element) can be monitored to indicate if the behavioral principles are being administered properly and are therefore often examined as more proximal measures of usability and the delivery of behavioral strategies [25]. Use data are often the criteria used to trigger considerations of changes to the BIT.

The TIPs framework can be viewed through the lens of mediational models in which an intervention is intended to affect intermediary outputs and outcomes [26]. The first part of the mediational model predicts that use of the BIT will increase the use, behavior, or experience defined by the principles. The second part of the mediational model predicts that the intervention principles will improve intervention outcomes. Thus, the principles and their measurement must remain fixed over the course of a trial. However, each new version of a BIT can be viewed as a moderator of this mediational model.

### Operationalizing Principles: The Principle Statement

The principles to be tested must be operationalized and stated a priori. This can be accomplished through a “Principle Statement” that describes each of the relevant BIT Model components [16] being evaluated, as shown in Figure 2. The formulation of a principle statement can be facilitated by using a model, such as the BIT Model, that integrates the conceptual and technological instantiation components. Using the BIT Model, a principle statement could take the following form: “The aim of the trial is to test an application that supports users in (*Behavioral Strategies*), delivered using (*BIT Elements, Characteristics, Workflow*) to improve (*Intervention Aim*)”. A principle statement might not contain all of these aspects, as some BIT elements, characteristics, or workflow may not be considered principles in the trial. A principle statement is needed to provide clarity to the aims, support change decisions during the trial (eg, defining what can and cannot be changed), as well as provide a method of documenting and reporting changes in a consistent manner.

As an illustration, a principle statement for MyFitnessPal a few years ago might have read “MyFitnessPal aims to support users in goal setting, self-monitoring, feedback using logging, and data reporting and vizualization features to support *weight loss and increased physical activity*”. This principle statement identifies the behavioral strategies and specifies a few BIT instantiation components linked to those strategies (ie, logging features for goal setting and monitoring, and data reporting and visualization to provide feedback on caloric intake and exercise), and measureable intervention aims (ie, weight loss and increased physical activity). In this statement, characteristics and workflow are not identified as principles being tested, thus, trialists would have been free to make changes to characteristics and workflow that did not substantially impact the behavioral strategies and BIT Model elements specified in the principle statement. MyFitnessPal’s addition of the feature allowing the use of barcode scanning for data entry can be seen as consistent with the principle statement, as it is a characteristic that simplified data logging elements. The addition of social network features, however, added substantial new functionality. A critical determination should be made as to whether social network features would alter the trial principles of goal setting, self-monitoring, and feedback and whether they expand beyond the behavioral strategies of goal setting and self-monitoring. One could imagine social networking features framed around these topics. However, social networking also adds other social motivators such as accountability [27], solicitation, and receipt of advice. These would likely constitute the addition of a behavioral strategy, thereby undermining the interpretability of trial results with respect to the principles being tested.

The principles may be defined broadly, focusing primarily on intervention aims and behavioral strategies, or very narrowly, including BIT elements, characteristics, and workflow. The maximal amount of definition would come much closer to a trial of a locked down BIT, while a definition that included no BIT instantiation features would be indistinguishable from an intervention that did not include technology. Thus, the inclusion of BIT instantiation features in a principle statement should be sufficient to define the intervention, but not include those areas that may allow for optimization in the service of testing the targeted principles.

### Making Changes During a Trial of Intervention Principles

Mid-trial changes to a BIT will frequently be the result of knowledge gained up until that point [6]. Trialists and developers may use various sources of information to trigger considerations of whether changes to a BIT during trials should be made, including use data, user feedback, and possibly intervention outcomes. Use data can include gross measures of use that can provide general information on whether the BIT is being used, as well as detailed use data from specific BIT elements that can indicate how they are being used. Such data can be collected and reviewed periodically. User feedback is also commonly captured both through self-report surveys as well as through interviews with users at the end of treatment or at specified points during treatment [28].

The decision to change a BIT is almost by definition triggered by observations that were not anticipated prior to the launch of the trial. Thus, the decision is based most often on investigator judgment. The decision to make a change is often not easy, as investigators are often balancing pressure to succeed and budget constraints in a situation where there is often considerable uncertainty. While clear plans for such unforeseen situations would be difficult to develop, planning nonetheless may help facilitate decision making under these circumstances.

Usually a decision to change a BIT based on observed data is made due to a belief that a specific behavioral strategy, BIT element, characteristic, or workflow is producing suboptimal usage or intervention outcomes. Implicitly, this decision is based on a comparison between an a priori expectation and observation of usage or effect. However, in practice, a priori expectations are often not clearly defined, which in effect leaves investigators making decisions based on what they believe they would have expected beforehand, rather than actual prior expectations. Thus, the decision-making process can be facilitated by making the implicit more explicit—defining the expected use of specific BIT elements and outcomes prior to the app launch. These a priori expectations can be informed by pilot testing, prior experience, published data, or consultations with advisors. The decision to make a change may also be informed by examining the relationship of the use of the element with outcomes (intervention and usage), bearing in mind that some outcomes, especially more distal primary outcomes, may change more slowly than more proximal intermediary outcomes tied to specific behavior change strategies [25].

### Degree of Blindness to Use and Outcome Data

Whether or not to keep investigators blind to some or all of the trial results during the trial and how to structure any lack of blinding are critical questions in managing TIPs. Investigator blindness is often thought to be a requirement for fixed interventions in order to protect against deliberate or unconscious biases. Full protection against this type of bias may be of less importance in quality improvement designs where there is a tacit if not explicit recognition that the BIT can be improved. There are situations where randomized trials explicitly use up-to-date outcome data to change certain aspects of a design (eg, adapting allocation ratios), but these must be used with caution to make appropriate causal inferences [29]. Also, it is possible to consider quality improvement changes in a BIT as discrete rather than continuous, with each instantiation being tested in one of a sequence of subtrials where blindness ends after each subtrial (see below under Evaluation). Alternatively, there are mechanisms for maintaining a degree of blindness throughout. In some cases, an investigator might have little involvement in the intervention being conducted and the eventual analysis of primary outcome data. In such cases, an unblinded BIT investigator, with blinded data collectors, analysts, and allocation concealment, has little potential to bias the research, short of engaging in research misconduct and might be the best person suited to review data to determine whether it is necessary to make changes and what those changes might be. Data from the control arms may be sequestered, thereby allowing investigators to monitor the experiences of BIT users but preventing them from knowing the results of the trial. This would allow for enough information to make decisions regarding changes but under the right controls could protect the evaluation from investigator bias.

### Decision Making

Making in-trial changes to a BIT carries a number of risks and should be undertaken only when deficiencies are observed. The goals of making changes are to improve the BIT’s capacity to achieve the primary intervention goals of the intervention using the intervention principles being tested, to prevent obsolescence of the BIT that may occur in the changing technological and application environment, and to maximize the amount of knowledge obtained from the substantial costs and efforts put into a trial. Bug fixes are probably the least controversial types of changes that can and are made during trials. Bug fixes are problems in the functionality of the BIT that might arise from changes in the technological environment (eg, changes in operating systems that cause problems for an app) and problems encountered in wider deployment not uncovered during initial testing. Other changes, however, implemented to improve functionality and usability open additional risks that can undermine TIPs. These risks can occur in at least three ways.

1. The proposed changes may directly diminish the BIT’s ability to provide the intervention principles by reducing its usability or usefulness. While investigators and developers would not likely consider such deleterious changes, this may occur inadvertently. The example noted above (the addition of reminder notifications) may increase initial response to prompts and engagement; however, this may also engage people who do not sustain engagement, thereby decreasing overall retention [30]. Therefore, researchers should carefully consider unintended consequences of a supposed improvement and intensively monitor potential changes resulting from this improvement to insure that it has improved the intervention.
2. Changes to a BIT may enhance components of the app that provide an alternative pathway to the intervention aims that undermines the primary principles being tested. For example, adding a motivational messaging element to an app being used to test the value of goal setting and feedback could introduce another behavioral strategy that would undermine the trial’s interpretability. Furthermore, the impact of changes may not result from a single decision but may be the consequence of a series of incremental changes that each by itself do no harm, but which in their totality undermine the trial.
3. A change might increase variability in how people use and benefit from a BIT. For example, in a BIT to increase physical activity through passive sensing and monitoring, developers might introduce a leaderboard that provides feedback about how one’s steps compares to other similar users. Leaderboards tend to be a polarizing feature, with some people motivated by such competition and therefore increasing the target behavior, while other people are negatively impacted (eg, experiencing negative emotions or loss of intrinsic motivation) thus decreasing use of the BIT and the target behavior [31]. In such a case, the BIT could still have the same impact for the “average” user, but as users either increase or decrease their use and activity in response to the feature, this would increase the variability of response. Increased variability in outcome could make it more likely that the null hypothesis (of no difference between groups) would be supported.

To prevent such problems, a clear set of procedures should be employed in making such decisions. First, the investigators should consider how the changes would affect the interpretation of the results of the trial. Most importantly, they should consider whether or not the proposed changes would open alternative explanations to any trial results. Second, any proposed changes should not just be considered in isolation but in the totality of all changes made to date. That is, the proposed changes would impact the interpretability of the results relative not only to the previous iteration, but also to the first deployed version of the BIT.

A number of questions can be considered when weighing the pros and cons of making an in-trial change to a BIT:

- Does the change interfere with a primary principle being tested?
- Would the proposed change alter the principle statement?
- What is the operational definition of success for a proposed change? Often the definition of success can be defined by the usage aims, which can be observed in the short-to-intermediate term. However, success may also be defined with respect to the intervention aims, which are more relevant but frequently require longer follow-up.
- If the introduction of the change is successful, would it create an alternative explanation for the success of the trial?
- If an alternative explanation is possible, are there methods that can be used that can reasonably eliminate that alternative explanation?
- If a change is not made, how would this impact the generalizability/implementation of findings from the trial?

In making a decision to change a BIT during a trial, input from a variety of perspectives is important to ensure that all possible impacts are considered. The areas of domain expertise brought to bear on the question may include behavioral science and theory, clinical or public health expertise, human factors engineering, trial methodology, statistics, and any other relevant area. It may also be useful to have guidance from knowledgeable people who are not part of the core investigative team, who may be able to see potential problems that a team, involved in the day-to-day operations, is less likely to see. If a panel of stakeholders, including potential users, were created to provide feedback during BIT design, this panel could be consulted on changes considered during the trial as well. Some decision-making functions could be embedded in the data safety monitoring board, if this board can operate in an efficient manner.

Once possible threats of a proposed change have been identified, methods of mitigating those threats to interpretability can be considered. For instance, using our previous example, if motivational messaging is added to an app with the intention of improving use, the impact of such a decision on a trial might be mitigated by adding identical or similar messaging to a BIT used in a control condition. Furthermore, subsequent investigations might be useful to demonstrate that a proposed principle is generalizable and persists when aspects not related to that principle are modified.

### Documentation and Reporting

Documentation is critical to TIPs of evolving technologies both to support the investigative team in decision making and in creating transparency. The principles being tested should be defined before the start of the trial, for example, using a principle statement. In general, the greater the specificity of the principle statement, the less latitude the investigator and developer will have in making changes.

Finally, documentation of the changes and the reasoning behind them will provide transparency and allow consumers of trial data to understand and accurately interpret the meaning of the trial results. While providing an accurate definition of the intervention in any peer-reviewed publication is important [32], reporting should also include changes made to BIT elements, characteristics, and workflow (and behavioral strategies if necessary) over the course of the trial, in accordance with the CONSORT-EHEALTH Guidelines [33].

We would like to emphasize, that in most cases, changes to BITs are relatively minor and infrequent. The cost of making continual or substantial changes to BITs is often a significant limiting factor. In addition, collecting, collating, and reviewing data that would drive these changes is time intensive and will also limit the frequency of such changes. Even without these barriers, researchers should be careful not to “churn” or overcorrect when making changes to the intervention elements, characteristics, or workflows. As easily implemented improvements are generated, these should be considered carefully, grouped into meaningful sets, and implemented as a set with appropriate version control of the software.

### Evaluation

#### Overview

The evaluation of RCT data on the efficacy or effectiveness of BIT in its entirety as an application can be performed using standard statistical analyses such as paired *t* tests or more complicated analytical procedures such as mixed effects modeling. BIT use can be reported using simple descriptive statistics such as the mean or media of app launches or site logins, cleaned for artifacts, and between-application comparison can also be performed in an unbiased fashion using comparative tests such as *t* tests, Mann-Whitney U tests, or mixed effects modeling. The advantage of evaluating the efficacy of a BIT as an application is its simplicity and usefulness for between-BIT comparisons. However, a standard analytic approach ignoring changes made to a BIT essentially aggregates the effects of those changes over time, combining the effects of the first version with the results of each of the subsequent iterations.

#### Verification of Improvements

An important part of any quality improvement effort is the verification that the changes made to the BIT had the intended effects. This verification can be performed both on the intervention outcomes and the use data. In most cases, such analyses are more likely to be better powered for use data than for primary outcomes, as use data are usually more proximal to the changes in BITs intended to improve usability. This can be evaluated by comparing use data (or outcomes) across different versions of the app or by testing the fixed effect of a time-varying ordinal component denoting the version in a longitudinal design. Such evaluations would need to check for changes in the sample (violations of the assumption of stationarity) that might occur over time. If changes in the recruited sample occurred over time, these changes would need to be addressed using covariates in the analysis or correcting through a propensity score analysis [34].

#### Optimization Trials for Local Evaluation

In practice, when a team has reasonable certainty on an approach to take, a specific change in the technology can be made, and the effect of that change can be monitored, as described above. However, it is not uncommon that development teams have more than one viable change and do not have enough information to make a decision. In such cases, optimization trials may be conducted within the treatment arm. The goal of an optimization trial is to maximize the outcomes of the trial participants, strengthen the test of the principles being evaluated, and support learning during the trial. A simple approach would be to randomize participants to versions of the BIT that contain the different solutions until the developers are confident that one of the solutions is superior.

Adequate power to detect differences would in many cases require large effect sizes under a traditional accuracy-centric framework that controls the study accuracy via type I error rate. This would normally not be possible for clinical outcomes, where sample sizes are determined by power analyses for the entire trial and may be a challenge even for use data (although those effect sizes should be larger if one of the options being tested is clearly superior). However, if optimization of a BIT is considered a local evaluation, within the trial, to support maximization of BIT use and/or outcomes of the trial participants, local trials and a selection paradigm may be more appropriate [35]. In this framework, assuming prior knowledge does not indicate a clear preference. A decision critical value may be defined with respect to a 50% type I error rate (ie, requiring *P* value <.50), thus imposing substantially smaller sample sizes. The one caveat is that the knowledge acquired from the decisions are intended for local optimization and cannot be generalized beyond the specific test of the application and should be used only for validating the effects of optimization efforts within the deployment for that trial.

#### Trials Involving Individual Level Adaptation Strategies

An important characteristic of BITs is that they can learn and respond to individual use patterns and self-reports or sensors of certain health outcomes. One could adopt outcome-adaptive randomization that uses the data from patients previously treated in the trial to tilt the randomization probabilities in favor of the instantiation components (elements, characteristics, and workflow) having comparatively superior outcomes such as maximum usage [36]. This adaptive randomization provides a balance between ethical concerns and the learning objective. Importantly, the adaptive randomization probabilities can be personalized based on a patient’s covariate or profile [37]. While statistical inferences (eg, hypothesis testing) after an outcome-adaptive procedure should be interpreted with caution, adaptive randomization is useful for optimizing in-trial outcomes and for selection purposes. In order for the adaptation to come into effect, we need to assign initial patients in a non-adaptive fashion (such as balanced randomization) so that we use the outcome data in these initial patients to change the randomization probabilities. However, the expected outcomes of patients will improve over time as the trial continues, and accrual and adaptation begin. In addition, in order to shorten the wait period before adaptation begins, adaptation can be made on the basis of early outcome or usage data that are indicative of the eventual outcome [38].

In the context of TIPs, randomization and treatment assignments can be made multiple times within each patient as in a crossover study design. Furthermore, the reassignment can be made based on the intermediate outcome or usage data as in a sequential multiple assignment randomized trial (SMART) design [39,40]. For example, suppose a patient is first assigned to receive no reminder and has not used the BIT for 2 weeks. Then the patient is reassigned to receive daily reminders and begins to check in to the app in response to the reminder. Then the frequency may be reduced to three times a week so as to avoid “overdose”. Generally, the data structure from a SMART design is dynamic in that the interventions are dependent on the intermediate response. For this type of data, we could use reinforcement learning techniques such as Q-learning to identify the optimal dynamic sequence of actions [41]. Q-learning involves fitting a regression model for a proxy of the eventual outcome at each decision stage so as to assess the impact of change and its interaction with the patient’s history (such as profile and intermediate response), and using backward induction to obtain the stage-wise optimal decisions. By virtue of randomization in a SMART, such analysis provides unbiased evaluation of the components and principles within a BIT. Importantly, on the basis of the evaluation, we could assess the optimal implementation of a BIT using backward induction.

To evaluate the effects of individual intervention components in complex interventions such as a BIT, n-of-1 trials can also be useful [42], in particular when the effects are stationary over time but heterogeneous across patients. While both n-of-1 trials and SMART involve re-randomization within a patient, their objectives are different. In a SMART, the goal is to identify a *sequence* of intervention components so as to maximize an eventual health outcome; in an n-of-1 trial, the goal is to identify an *intervention component* that is effective in a particular patient. These two designs provide complementary tools in the evaluation of BITs.

#### Evaluating Principles

The strongest test of a principle is to withhold it from some subjects and evaluate whether there is a difference in outcomes between those who received that principle and those who did not. Most BITs, however, rely on multiple behavioral strategies. One can evaluate the behavioral strategies as a package, much as is commonly done in trials of behavioral and psychological interventions. Alternatively, trials using multiphase optimization strategies and factorial designs can be used to test a number of principles in a single trial [43].

For trials evaluating multiple principles as a package, post hoc evaluations may be performed to test the relative contribution of different components to outcomes. For example, if an app relies on goal setting, monitoring, and feedback as change principles, one could evaluate the relative use of each of the components to outcome. Such designs are purely correlational and are therefore not conclusive, but they may provide information that could be valuable to future investigations.

#### Development of Statistical Methodology for Trials of Intervention Principles

While many statistical methods are available that can be used for the evaluation of TIPs, approaches that truly integrate optimization methods and RCT analysis will be required. Methodology used for cumulative trials may be useful in this context. A “cumulative trial” is a sequence of smaller trials testing single or multiple adaptations in the technology that can be analyzed in a combined fashion relative to a control condition [29,44]. In the context of TIPs, a cumulative trial could involve a sequence of discrete quality improvement tests, intended to improve BIT performance, compared against a single control condition that can test underlying principles. This proposed method evaluates the effect of the BIT on the intervention outcome, the comparative effect of individual BIT versions, and the effects of varying levels of use of the BIT on outcomes across and within BIT versions.

As an example of a potential cumulative TIP design, we consider how such a design might have been used during the early period of MyFitnessPal. This proposed TIP design would model the underlying intervention impact of the principle as a function of the effectiveness of the BIT to affect outcomes. In symbols, we consider a sequence of trials, *t*=1 , …, T, each of which is testing a control condition against one or more versions of active BITs. For simplicity, suppose there is a single active version that is modified in each new trial *t*. Each individual *i* in trial *t* is randomly assigned to either active intervention, where *Z*_*it*_=1 or control *Z*_*it*_=0. For each of the trials, we suppose there is a common use metric, that is, *U*_*it*_,=1 if person *i* achieves full usage, 0 if no usage, and between these extremes for partial usage. In this case, usage would include the degree of reporting of meals and exercise. Let *Y*_*it*_ be the measured health outcome for subject *i* in trial *t*; in this case it would be weight loss. The effect of the active version compared against control could be modeled as involving three components in a multiplicative model. The first component involves a factor whose strength is measured by the parameter β that compares the BIT versus control under a condition where the subject receives the optimal version and the individual has complete usage. The second component’s strength is measured by a parameter γ_t_ for all other versions that accounts for their being suboptimal. This component, given within the first brackets below, is bounded between zero and one. The third component, parameterized by δ, accounts for degradation in effects due to incomplete usage by the individual. BIT use can be calibrated on a zero to one scale using simple descriptive statistics such as the mean or median of app launches or site logins, cleaned for artifacts, and controls would receive a zero value unless they self-expose to an alternative BIT. This component is the third bracketed expression below, and it too is bounded between zero and one:

*Y*_*it*_=µ + *Z*_*it*_ β * [exp(γ_t_) / 1+ exp(γ_t_)] * [exp(*U*_*it*_ δ) / 1+ exp(*U*_*it*_ δ)] + ε_*it*_ (1)

The last term in equation (1), ε_*it*_ *,* corresponds to an error term_._ We can interpret β as the optimal effect of a BIT against control on the intervention outcome; the γ_t_ represents how close to optimal this version is, and δ can be interpreted as the sensitivity that usage or dosage has on the health outcome. The degree of usage itself can be modeled as well, such as:

log (*U*_*it*_ / 1 + *U*_*it*_ )= θ + υ_t_ + ε’_*it*_ (2)

In this modeling equation (2), the value of υ_t_ represents the average usage on this transformed scaled for the *t* ^th^ version. These models are illustrations of how suboptimality and usage can be included and would likely need refining so that they provide a good fit to the data from a sequence of trials. Statistical inference across trials would need to account for trial level variation, much like meta-analysis, network meta-analysis, and other synthesis methods include trial level random effects [44]. In addition, individual level factors would need to be included both as main effects and potential moderator variables. Designs that deliberately look at variation in impact as a function of baseline participant characteristics are useful for examining this moderation of effects on both use and intervention outcomes. To ensure sufficient power to detect moderation, participants are often stratified by baseline characteristics (eg, age) and then randomized within these strata.

Equations 1 and 2 can be used to conduct a type of mediation analysis that partitions how much of the impact is explained through usage. Importantly, random effects can be entered into equation (1) to account for imperfect specification of a hypothesized mediation pathway. As this equation stands without random effects, all the effects of the intervention are required to flow through a specific mediation model (eg, usage). Incorporation of random effects provides the opportunity to address more complex explanations of an intervention’s effect. Also, classic mediation modeling [26] can be used to test whether an intervention’s impact on clinical outcomes is due to the use of the principles underlying the BIT. Such mediation analyses are most useful when two versions of a BIT are tested against each other. A mediation analysis in a head-to-head trial of two BITs would first test whether use of the BIT varies, then test whether variation in use affects the more distal health outcome.

## Discussion

### Principal Considerations

Developers frequently make changes and adjustments to BITs during the course of trials for numerous reasons. Often original specifications are created with insufficient information. Even after careful usability and field testing, problems can be uncovered during broader deployment in a trial. Locking down an intervention during a trial, thereby forcing investigators to test a BIT with known deficiencies, decreases the likelihood that useful knowledge will come from the trial. Furthermore, the resulting BIT may not be appropriate for the technological environment that exists when the trial is completed. Thus, integrating quality improvement into RCTs can spur innovation, allow for BITs to evolve over time, and improve the likelihood that useful information will result from the trial.

We propose that these trials be conceptualized as evaluations of principles rather than of BITs themselves. As BITs, unlike pharmaceutical agents, do not stay static over time, the principles being evaluated represent the more static and generalizable component of the trial. This is valuable as the current form of any BIT at any given time will likely be relatively short lived. These trials, which we call TIPs, would evaluate constructs that could be more broadly applied, thereby moving the field ahead more rapidly.

We have presented an initial proposal of a method of defining the principles being evaluated that incorporates the essential clinical features, including the clinical aims and behavioral strategies, and the technical features, including BIT elements, characteristics, and workflow. This principle statement defines what is being tested, thereby promoting clarity for the research team and communication of aims to consumers of trial information. The principle statement can also support investigators in decision making around in-trial changes to the BIT. Changes in seemingly unrelated elements, however, may still have secondary effects on the principles under evaluation. Careful consideration of these potential secondary impacts is required to preserve the interpretability of the results. We recognize that even with these procedures, the risk of damaging a trial through in-trial changes to a BIT cannot be completely eliminated. However, we argue that the potential risks are far less than the more certain risks of persisting in a trial with a BIT that is underperforming in critical areas.

This approach fits well with changes in many funding agencies where there is a growing emphasis on trials that focus on mechanisms that can be more broadly applied [45]. Industry often has needs that are different from academic researchers, in that they are seeking to acquire data to support the effectiveness of their BITs for marketing purposes. But there too, the BIT that is purchased at one point is likely to change over time. A clear definition of the principles tested would allow the purchasers of BITs to have a clear definition of the parameters of the BIT they are purchasing, thereby allowing them to determine when the BIT they are receiving no longer meets the specifications of the BIT they purchased.

This is by no means the first proposal to enhance the usual RCT design to address the needs of BIT evaluation. Multiphase Optimization Strategy trials (MOST) have become an accepted method of using fractional factorial designs to evaluate and optimize the components of BITs [39]. SMART designs allow for the evaluation of systems that provide different components at various times depending on specified criteria [39,40] and thus have considerable value in investigating questions such as workflow. Continuous evaluation of evolving behavioral intervention technologies (CEEBIT) is a proposed method of comparatively evaluating BITs in local settings, a form of post-marketing surveillance allowing organizations providing BITs to continuously evaluate their usefulness in among their consumers [5]. TIPs extend this process of RCT innovation by providing a methodology for harnessing knowledge of BITs gained during the trial and managing changes in BIT specifications. TIP methodology is intended to prevent such changes from damaging the interpretability of results, while maximizing the amount of knowledge gained. Additional work might be undertaken after a principled trial to ensure the generalizability of the principles tested. However, these steps should be seen after initial trials provide sufficient evidence that these principles are being closely tied to behavior change.

TIP methodology is not intended as a substitute for careful usability and field testing prior to a trial. Ensuring that a BIT is usable, both through laboratory and field testing, is an essential step in acquiring information, refining the BIT, and ensuring that the tools are usable and useful for their intended consumers. Furthermore, information gained from laboratory testing cannot be obtained easily in field deployment. Early field testing can uncover fundamental problems that reduce the effectiveness of the BIT in promoting the intended behavioral strategies. Indeed, initiating a trial with a poorly developed app greatly increases the risk that a trial will fail, since early users will be less likely to show benefit, which will decrease the trial’s power to see changes in the primary outcomes. Additionally, adopting TIPs in practice is not without its challenges. We have used the BIT Model [16] to identify levels and elements contained within a BIT and to emphasize the common behavioral strategies, the conceptual “how” of a BIT, used to attain the intervention goals, but other models could also be applied within the TIP framework. The underlying notion, however, that intervention trials are truly testing common elements rather than specific instantiations, has been emphasized with other models [13-15,19,20], and we have highlighted considerations on how to make use of these conceptual notions within trials of BITs. We believe adoption of TIPs is an initial step to increase the transparency of the evaluation process for technology-based interventions and improve the value of the information gleaned from these trials. While further work is needed, this initial framework provides a common language and practices for the field to consider.

### Conclusion

To the best of our knowledge, this is the first methodology proposed to manage changes to a BIT during the course of a trial. The proposed methods are intended to ensure that the principles under evaluation are preserved and even enhanced by allowing learning and optimization to occur during the trial.

## Abbreviations

BIT: behavioral intervention technology
RCT: randomized controlled trial
SMART: sequential multiple assignment randomized trial
TIP: trial of intervention principles
